# Aurora-C Interactions with Survivin and INCENP Reveal Shared and Distinct Features Compared with Aurora-B Chromosome Passenger Protein Complex

**DOI:** 10.1371/journal.pone.0157305

**Published:** 2016-06-22

**Authors:** Kaori Sasai, Hiroshi Katayama, David H. Hawke, Subrata Sen

**Affiliations:** 1 Department of Translational Molecular Pathology, University of Texas M.D. Anderson Cancer Center, Houston, Texas, United States of America; 2 Department of Systems Biology, University of Texas M.D. Anderson Cancer Center, Houston, Texas, United States of America; Virginia Tech, UNITED STATES

## Abstract

Aurora-C, a member of the Aurora kinase family that can complement Aurora-B function in mitosis is either moderately expressed or repressed in most adult somatic tissues but is active in early embryonic development and expressed at elevated levels in multiple human cancers. Aurora-C overexpression reportedly plays a role in tumorigenic transformation. We performed detailed characterization of Aurora-C interactions with members of the Chromosome Passenger Complex (CPC), Survivin and Inner Centromere Protein (INCENP) in reference to known Aurora-B interactions to understand the functional significance of Aurora-C overexpression in human cancer cells. The results revealed that silencing of Aurora-C or -B individually does not affect localization of the other kinase and the two kinases exist predominantly in independent complexes *in vivo*. Presence of Aurora-C and -B in molecular complexes of varying as well as overlapping sizes and co-existence in INCENP overexpressing cells indicated oligomerization of ternary complexes under different physiological conditions *in vivo*. Furthermore, Aurora-C and -B stabilized INCENP through interaction with and phosphorylation of the IN box domain while Aurora-C was activated following Survivin phosphorylation on Serine 20. Phosphorylation of Survivin residue Serine 20 by Aurora-C and –B appears important for proper chromosome segregation. Taken together, our study suggests that Aurora-C, expressed at low levels in somatic cells, functions as a catalytic component of the CPC together with Aurora-B through mitosis. Elevated expression of Aurora-C in cancer cells alters the structural and functional characteristics of the Aurora-B-CPC leading to chromosomal instability.

## Introduction

Aurora kinase family proteins have been conserved through evolution as critical regulators of cell division. While the genomes of fission and budding yeast encode a single Aurora kinase, Ipl1 and Ark1 respectively, larger metazoan genomes express up to three members of the kinase family. Two paralogues, Aurora kinase-A and -B (hereafter referred to as Aurora-A and Aurora-B) have been identified in *Drosophila melanogaster*, *Caenorhabditis elegans* and *Xenopus* while a third member of the family, Aurora kinase-C (referred to as Aurora-C) has been detected only in mammals [1]. A number of studies on Aurora-A and Aurora-B have revealed that these two enzymes predominantly localize to discrete mitotic structures and coordinately regulate progression of cells from G2 through cytokinesis [2].

Aurora-B kinase activity, required for proper chromosome alignment, segregation, and cytokinesis is regulated through interactions with three non-enzymatic proteins, Inner centromere protein (INCENP), Borealin/Dasra B, and Survivin, which are also involved in targeting the kinase to its subcellular localizations [3]. Aurora-B, INCENP, Borealin, and Survivin, exist in a conserved complex, referred to as chromosomal passenger complex (CPC), which displays a dynamic localization pattern during mitosis, appearing first along the chromosome arms followed by localization at the inner centromeres from prophase through metaphase, and then re-localization at the spindle midzone and midbody during anaphase through cytokinesis. Characterizations of intermolecular complexes have led to the suggestion that Aurora-B may exist in two separate complexes, a holo-complex containing Borealin, INCENP, Survivin and another sub-complex with INCENP only [4]. Inactivation of Aurora-B and loss of CPC subunits impair error correction of kinetochore-microtubule attachments and cytokinesis [5, 6].

Aurora-C, first identified in mouse sperms and oocytes, is expressed at significantly higher levels in testes compared to other somatic tissues [7] and the *AURKC* gene was initially reported to be functionally involved in male meiotic division in mouse [8]. These findings were subsequently validated with *AURKC* null male mice reported to be viable but sterile [9]. In humans, a single nucleotide deletion in *AURKC* coding region was shown to result in polyploid spermatozoa and male infertility [10]. Interestingly, Aurora-C was also found to complement Aurora-B function in mitotic cells and identified as a CPC member [11, 12], with a later study reporting wild type Aurora-C rescuing normal mitotic functions in Aurora-B deficient HeLa cells [13]. More recent findings on the role of Aurora-C in the CPC during human pre-implantation embryo development [14] and early embryonic development of mouse [15] have validated that Aurora-C can function as a catalytic component of CPC at certain physiological stages of somatic cell development. These observations together with the published reports on gene amplification-driven overexpression of Aurora-C in breast cancer cells [16] and elevated expression inducing cell transformation *in vitro* and tumor formation *in vivo* [17] suggest functional involvement of Aurora-C overexpression in malignant transformation of somatic cells. Furthermore, Aurora-C was found to co-localize with a breast cancer susceptibility gene, *TACC1* (transforming, acidic coiled-coil containing protein 1) in the midbody of HeLa cells during cytokinesis and phosphorylate Serine 228 of TACC1 [18], indicating a possible TACC1 mediated role in the cellular transformation process. In addition, Aurora-C expression was shown to be involved in TNFα induced chromosomal instability in response to inflammatory signals [19].

In view of overlapping and complementing CPC associated functions of Aurora-C and -B reported in somatic cells with studies also suggesting the two kinases co-existing in the same complex [12, 20], we performed a detailed characterization of Aurora-C interactions with Survivin and INCENP in reference to Aurora-B utilizing human cancer cell lines naturally overexpressing endogenous Aurora-C as well as with WT and mutant recombinant proteins expressed *in vitro* and *in vivo*. Results reveal that endogenous Aurora-C, similar to Aurora-B, binds directly to and co-localizes with Survivin. Aurora-C binding with Survivin and localization depends on a C-terminal sequence spanning eighteen amino acids conserved in Aurora-B. INCENP and Survivin regulate proper localization of Aurora-C as is the case with Aurora-B although exclusive loss of either Aurora-C or -B kinase does not affect localization of the other. Interestingly, our data also demonstrate that unlike what has been reported earlier [12, 20], Aurora-C and -B predominantly exist in separate complexes with INCENP and Survivin *in vivo*. The two kinases, however, are detected in the same complex in presence of high INCENP levels, possibly due to oligomerization of ternary complexes. Finally, we mapped a novel site of phosphorylation on Serine 20 of Survivin by Aurora-C and -B that is involved in the activation of Aurora-C kinase. Survivin phosphorylation on Serine 20 plays an essential role in proper chromosome alignment and cytokinesis.

## Materials and Methods

All the materials used in the study were verified for authenticity with appropriate analytical techniques and the experimental methods were repeated at least two times or more to ensure reproducibility of the results being reported.

### Plasmid Construct

To obtain human Aurora-C cDNA have been described previously [11]. Human Aurora-B cDNA and human Survivin cDNA were obtained by polymerase chain reaction (PCR) using total RNA from HeLa cells. A human INCENP cDNA was subcloned into HA tagged pcDNA3 vector [11] by using pBluescript SK (-) INCENP [5]. Aurora-C N-terminus (aa 1–40) and C-terminus (aa 41–309; kinase domain, aa 41–291, aa 41–280, aa 41–250, aa 41–220, aa 41–190, aa 41–160 and aa 41–130) deletion mutants were amplified by PCR and subcloned into pGEX4T-1 vector (GE Healthcare) and pcDNA 3.1 His vector (Thermo Fisher Scientific). ΔINCENP deletion mutants, C3 (aa 822–919), C4 (aa 855–919), C5 (aa 878–919), C6 (aa 822–897), C7 (822–877) and C8 (aa 822–892), which were described previously [5], were subcloned into the pGEX4T-1 vector. For baculoviral constructs, Aurora-C and Survivin cDNAs were subcloned into modified pVL1393 vector (BD Biosciences), which provides an N-terminal glutathione *S*-transferase (GST) tag. The pHI100-GST-Aurora-B WT and KD, pHI100-His-INCENP full-length WT and 3A (T893A, S894A, S895A), ΔINCENP C3 (aa 822–919) WT and 3A (T893A, S894A, S895A) were described previously [5]. All point mutants were obtained by using the QuikChange Site-Directed Mutagenesis kit (Agilent Technologies) according to the manufacturer’s protocol.

### Expression and Purification of GST and His Tagged Proteins

Bacterial fusion proteins were produced in BL21 pLys bacteria according to the manufacturer’s protocol (GE Healthcare). For insect cells produced GST or His tagged proteins, Sf-9 cells were infected with appropriate combination or individual baculovirus. The complexes and individually produced proteins were purified with glutathione sepharose or Ni-NTA agarose as described previously [5]. For *in vitro* reconstitution kinase assay, GST-Aurora-C and GST-Aurora-B proteins were eluted by using B-PER GST Spin Purification kit (Thermo Fisher Scientific). His-INCENP proteins were eluted with Elution buffer (50 mM NaH_2_PO_4_, 300 mM NaCl, 250 mM imidazole). Eluted proteins were dialyzed in dialysis buffer (50 mM Tris pH 7.5, 15 mM MgCl_2_, 1 mM DTT and 1 mM PMSF). The fusion proteins were used for *in vitro* binding assay and *in vitro* kinase assay.

### *In Vitro* Kinase Assay and *In Vitro* Binding Assay

For *in vitro* kinase assay, immune complexes were washed 4 times with L buffer containing 500 mM NaCl (1% NP-40, 500 mM NaCl, 5 mM EDTA, 50 mM NaF, 20 mM Tris pH 7.5, 1 mM Na_3_VO_4_, 10 μM Na_2_MnO_4_, 1 mM PMSF, 1 μM Microcystin LR, 0.2 μM okadaic acid, Protease inhibitor cocktail (Roche)) and then washed twice with kinase buffer (50 mM Tris pH 7.5, 15 mM MgCl_2_, 2 mM EGTA, 0.5 mM Na_3_VO_4_, 1 mM DTT). Kinase reactions were performed for 30 min at 30°C in kinase buffer containing 1 μg histone H3 (Merck Millipore), 50 μM ATP, and 5 μCi of [γ- ^32^P] ATP. The reaction was terminated by adding SDS sample buffer and resolved by SDS-PAGE.

Survivin or Aurora-C proteins were produced using TNT T7 Quick Coupled Transcription/Translation system (Promega) in the presence of ^35^S-methionine. *In vitro* translated proteins were incubated with GST-Aurora-C, GST-Survivin, or GST-INCENP deletion mutants in NET-N buffer (50 mM Tris pH 8.0, 1 mM EDTA, 150 mM NaCl, 0.5% NP-40, 50 mM NaF, 2 mM Na_3_VO_4_, 1 mM PMSF, 1 μM Microcystin LR, Protease inhibitor cocktail) for 1.5 h at 4°C, then washed 3 times with NET-N buffer. The bound proteins were analyzed by SDS-PAGE.

### Cell Culture and Transfection

All cell lines were obtained from ATCC. 293T, PC-3 and SK-OV-3 cells were cultured in Dulbecco's modified Eagle's medium, RPMI1640 medium, and McCoy's 5a medium, respectively. Each medium was supplemented with 10% fetal bovine serum, streptomycin and penicillin. HeLa cells and Tet-on HeLa cells were cultured as described previously [11]. Sf-9 cells, insect cells, were cultured in TNM-FH Insect Medium (BD Biosciences) supplemented with 50 μg/ml gentamicin (Thermo Fisher Scientific). Plasmids were transfected by using Lipofectamine (Thermo Fisher Scientific). Twenty-four hour after transfection, cells were lysed in L buffer containing 250 mM NaCl.

For protein stability assays, 24 h after transfection, cells were trypsinized, split into several dishes, and then cultured for additional 24 h. Then cells were treated with 50 μM of protease inhibitor N-acetyl-L-leucyl-L-leucyl-L-norleucinal (LLnL) for 6 h.

To establish stable cells expressing wild type or mutant Survivin, HeLa cells were transfected with GFP-Survivin WT, S20A, or S20E mutants. Twenty-four hour after transfection, transfected cells were selected in neomycin (600 μg/ml) for 2 weeks and individual clones isolated.

### Immunoprecipitation and Immunoblot

0.5–1 mg of lysate were incubated at 4°C for 3 h or overnight with 1 μg of anti-Flag M2 monoclonal antibody (Sigma-Aldrich #F3165), anti-Flag M5 monoclonal antibody (Sigma-Aldrich #4042), anti-Living Colors Full-Length A.v. Peptide antibody (BD Biosciences #632377), anti-Aurora-C polyclonal antibody (Thermo Fisher Scientific #38–9400), anti-Survivin monoclonal antibody D-8 (Santa Cruz Biotechnology #sc-17779), anti-Survivin polyclonal antibody FL-142 (Santa Cruz Biotechnology # sc-10811), normal mouse IgG (Santa Cruz Biotechnology #sc-2025), or normal rabbit IgG (Santa Cruz Biotechnology #sc-2027). Immune complexes were incubated with 16 μl of protein G-agarose (Sigma-Aldrich) for 1 h. Samples were spun down and washed 4 times with L buffer containing 250 mM NaCl and resolved by SDS-PAGE.

Immunoblots were performed with the following primary antibodies: anti-Aurora-C polyclonal antibody (Thermo Fisher Scientific #38–9400), anti-Survivin monoclonal antibody D-8 (Santa Cruz Biotechnology #sc-17779), anti-Flag M2 monoclonal antibody (Sigma-Aldrich #F3165), anti-GFP monoclonal antibody (MBL #M048-3), anti-AIM-1 (Aurora-B) monoclonal antibody (BD Biosciences # 611082), anti-INCENP polyclonal antibody clone 55 [5], anti-Phospho-Aurora A (Thr288)/Aurora B (Thr232)/Aurora C (Thr198) (D13A11) XP polyclonal antibody (Cell Signaling Technology #2914), anti-Phospho-histone H3 (Ser10) (D2C8) XP polyclonal antibody (Cell Signaling Technology #3377), anti-histone H3 (D2B12) XP polyclonal antibody (Cell Signaling Technology #4620), anti-HA (Santa Cruz Biotechnology #sc-7392 and #sc-805, anti-His (Santa Cruz Biotechnology #sc-8036), anti-HSP90 (Santa Cruz Biotechnology #sc-13119).

### Short Interfering RNA (siRNA) Treatment

SiRNAs against Aurora-C [11], Aurora-B [21], INCENP [5], Survivin [22] were transfected by using Oligofectamine (Thermo Fisher Scientific). GL2 siRNA duplexes (GE Healthcare) were used as control. Cells were analyzed 48 h after transfection.

### Immunofluorescence Microscopy

Tet-on HeLa cells expressing GFP-Aurora-C grown on poly-L-lysine-coated coverslips were induced by addition of 1.5 μg/ml doxycycline (Dox) and treated with siRNA for 48 h. Immunostaining was performed as previously described [23] with anti-INCENP polyclonal antibody clone 55 (1:1,000) [5], anti-AIM-1 (Aurora-B) monoclonal antibody (1:300) (BD Biosciences # 611082), anti-Survivin monoclonal antibody D-8 (1:300) (Santa Cruz Biotechnology # sc-17779), or anti-α/β-tubulin (TU27B) antibody (1:300) [23]. FITC or Texas-Red conjugated secondary antibodies (Thermo Fisher Scientific # 31583 and T-862) were applied at 1:300 dilutions and DNA was counterstained with 4, 6-diamidino-2-phenylindole (DAPI).

### Mass Spectrometry

Protein samples were phosphorylated *in vitro* and incubated with PreScission protease (GE Healthcare) overnight at 4°C. The phosphorylated protein was separated by SDS-PAGE and stained with Coomassie Brilliant Blue (CBB). The band was excised, washed, destained, reduced, and alkylated with iodoacetamide and digested with sequencing-grade endoproteinase Lys-C (Wako Chemicals). The digest was desalted on a C18 Zip-Tip (Merck Millipore), mixed with matrix (ACHA), spotted onto a plate (dried-droplet method) and analyzed on a 4700 TofTof (Thermo Fisher Scientific).

### Sucrose Gradient Sedimentation

SK-OV-3 cells were treated with nocodazole (50 ng/ml) for 24 h and lysed with L buffer containing 150 mM NaCl. 2.5 mg of lysate were loaded onto 5–30% sucrose gradients and centrifuged at 40,000 rpm for 17 h at 4°C with a SW-55Ti rotor. As molecular weight markers, Gel Filtration Standard (BIO RAD) was run in a parallel gradient.

## Results

### Binding and Co-localization of Aurora-C with Survivin

**Fig 1:**
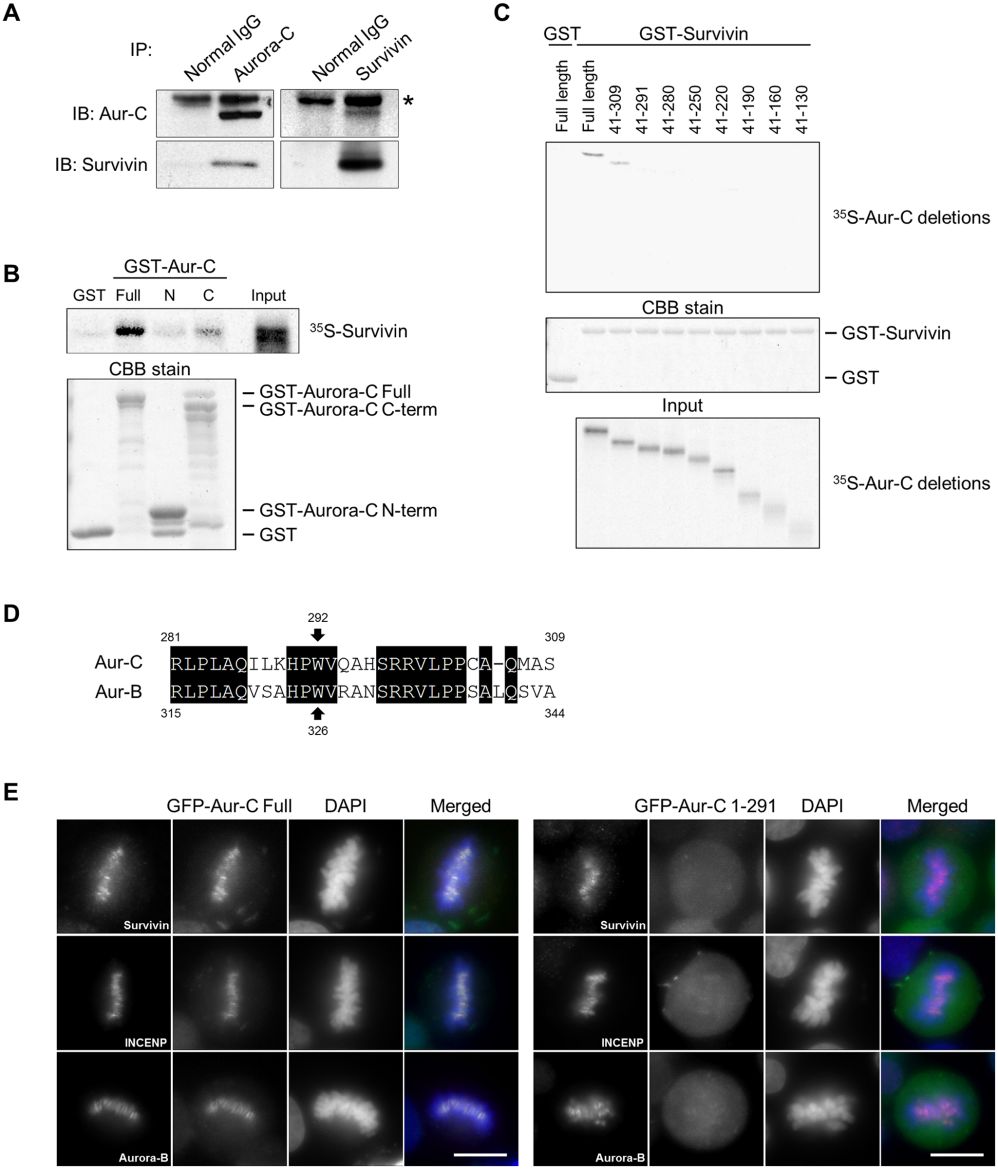
C-terminus end of Aurora-C binds with Survivin and is responsible for its localization. (A) PC3 cells were treated with 500 ng/ml nocodazole for 24 h and lysed with L buffer containing 250 mM NaCl. 1 mg of lysate was immunoprecipitated with 1 μg of anti-Aurora-C, anti-Survivin, or normal IgG antibodies respectively. Immunoprecipitates were immunoblotted with indicated antibodies. The asterisk indicates non-specific bands. (B) ^35^S-labeled Survivin was incubated with beads bound to any of the following: GST, GST-Aurora-C full-length (Full), N-terminus amino acids 1–40 (N), or C-terminus amino acids 41–309 (C). Beads were resolved by SDS-PAGE, and visualized by autoradiography (for binding, top) or CBB stained (bottom). (C) ^35^S-labeled Aurora-C full length or deletion mutants were incubated with beads bound to GST or GST-Survivin, and analyzed as in B. The panel shows autoradiography (for binding, top; for input, bottom) or CBB stained gel (middle). (D) Alignment of the C-terminus sequences of Aurora-C and Aurora-B. (E) Localization of GFP-Aurora-C full length or GFP-Aurora-C deletion mutant (amino acids 1–291) in HeLa cells. Transfected cells were immunostained with indicated antibodies (red). DNA was stained with DAPI (blue). Scale bars indicate 10 μm. Each experiment was repeated at least twice with two to three biological replicates and the figures show representative images.

While physiological interaction between Aurora-C and INCENP has been demonstrated, it remains to be investigated if endogenous Aurora-C and Survivin exist in a complex. To this end, we performed *in vivo* co-immunoprecipitation studies both in human cells naturally overexpressing endogenous Aurora-C and in cells co-expressing epitope tagged recombinant proteins. Endogenous protein interaction was investigated in the human prostate carcinoma cell line PC-3 and in the ovarian carcinoma cell line SK-OV-3, earlier reported by us to be overexpressing Aurora-C [11]. Immunoprecipitation of PC-3 cell lysates with respective antibodies in reciprocal experiments revealed the presence of Aurora-C and Survivin in the same immune complexes providing first evidence of interaction between the two endogenous proteins *in vivo* (Fig 1A). Similar results were seen in SK-OV-3 and MCF-10A, normal mammary epithelial cells (data not shown). To map the Aurora-C domain binding to Survivin, we carried out *in vitro* binding assays of ^35^S-methionine labeled Survivin with recombinant full length as well as truncated forms of Aurora-C representing the N-terminal (amino acids 1–40) and the C-terminal (amino acids 41–309) ends of the protein fused to GST. Direct binding of the full length and the C-terminal peptide (amino acids 41–309) of Aurora-C with Survivin was detected and specificity of this interaction was evident due to absence of any binding with GST alone or with the N-terminal forty amino acids of Aurora-C (Fig 1B). To further fine map the binding domain, a series of deletion mutants spanning different lengths of the C-terminal 289 amino acids (amino acids 41–309) were utilized for additional *in vitro* binding assays. We found that elimination of the most distal C-terminal eighteen amino acids of Aurora-C obliterated Survivin binding. Thus, none of the C-terminal deletion fragments containing amino acids 41–291 or less revealed any binding with GST-Survivin (Fig 1C), suggesting that the terminal eighteen amino acids at the C-terminal end of Aurora-C contain the motif responsible for binding with Survivin. These eighteen amino acids of Aurora-C share high homology with those of Aurora-B (Fig 1D) and include the eight amino acids (residues 326–333) implicated in Aurora-B localization and function [24]. The role of the C-terminal eighteen amino acid sequence of Aurora-C in its localization was then investigated by direct immunofluorescence analyses of GFP tagged full length and a deletion construct lacking these residues. Results revealed that while the full length protein co-localized with Survivin, INCENP, and Aurora-B around the centromeres of metaphase chromosomes, as expected, the deletion construct lacking the C-terminal eighteen residues was diffusely distributed in the cytosol (Fig 1E).

### Functional Interactions of Aurora-C and -B complexes

In order to investigate if catalytic activity of Aurora-C is necessary for proper localization of the CPC, we analyzed the subcellular distribution of CPC proteins in the presence of wild type (WT) and kinase dead (KD, K76R) form of GFP-Aurora-C in stable Tet-on HeLa cells. Since none of the 15 available antibodies worked optimally for immunofluorescence detection, we utilized GFP tagged Aurora-C for intracellular localization studies. The GFP-Aurora-C immunoprecipitated with anti-GFP antibody revealed the presence of Survivin and INCENP in the same immune complex (data not shown), thus verifying that the GFP tagged recombinant Aurora-C protein interactions *in vivo* mimic those detected for the endogenous protein shown in Fig 1A. While the WT protein was localized correctly as expected, the KD mutant showed near correct localization only in cells, which expressed low amounts of the protein. In cells expressing high amounts of the catalytically inactive KD form, Aurora-C was diffusely distributed in the cells (S1A Fig). In addition, endogenous INCENP, Survivin, and Aurora-B were also mis-localized in cells expressing high amounts of the Aurora-C KD mutant (S1B Fig). Similar mis-localization of INCENP and Survivin in the presence of inactive Aurora-B mutant was reported earlier [5, 25]. Our findings that mutant Aurora-C has a dominant negative effect on Aurora-B localization indicated that the two kinases may share a common phosphorylation substrate for proper localization of the CPC to the centromeres.

**Fig 2:**
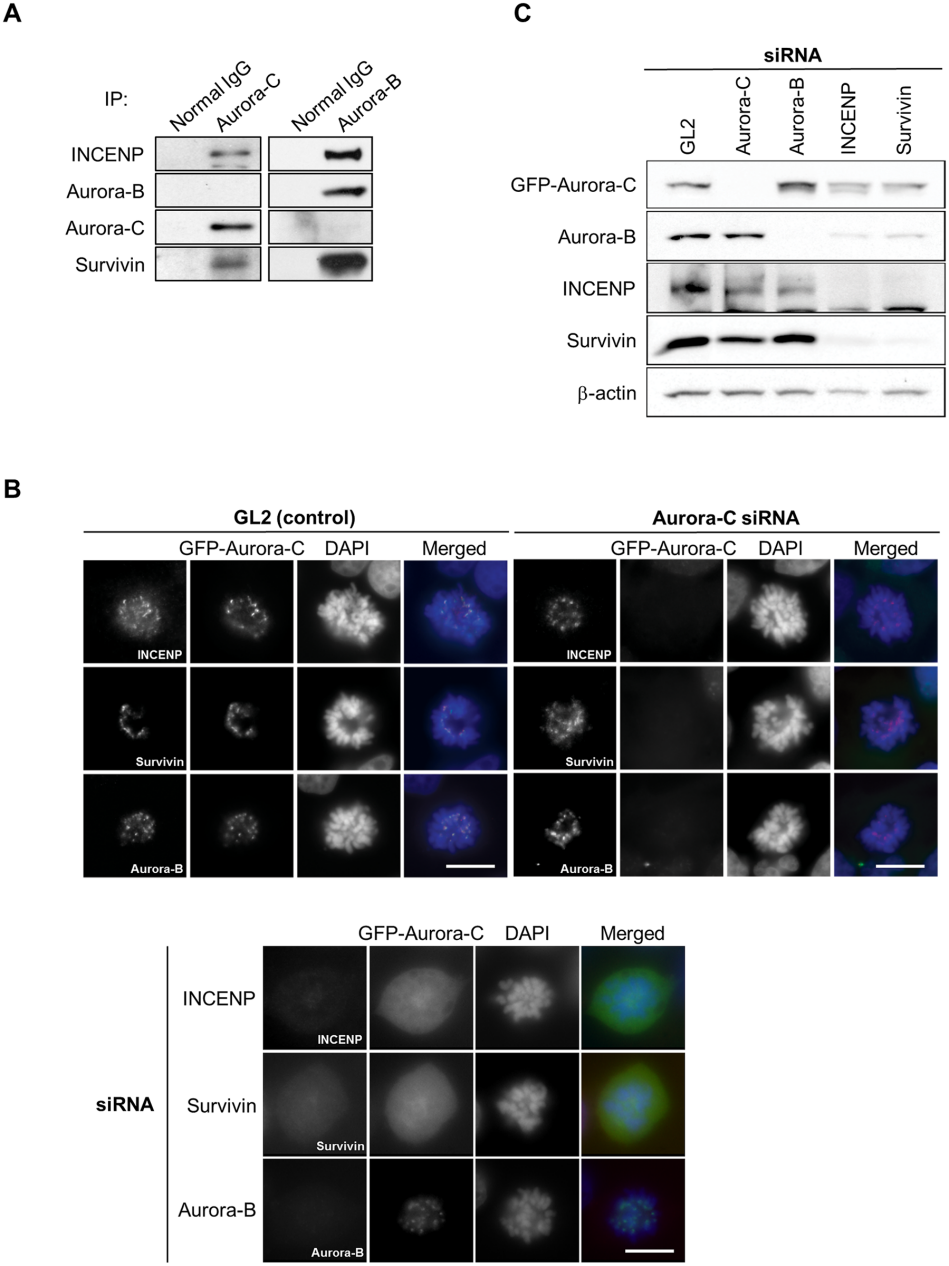
Localization following gene silencing and immunoprecipitation indicate independent Aurora-C and Aurora-B complexes. (A) Nocodazole (500 ng/ml) treated 1.7 mg of PC-3 cell lysate were immunoprecipitated with 2 μg of anti-Aurora-C and anti-Aurora-B antibodies. Immunoprecipitates were immunoblotted with the indicated antibodies. (B) GFP-Aurora-C expressing Tet-on HeLa cells was transfected with control siRNA (GL2) or with siRNA against Aurora-C, Aurora-B, INCENP, or Survivin for 48 h. The cells were immunostained with anti-INCENP (top), anti-Survivin (middle), or anti-Aurora-B (bottom) antibodies, respectively (red). DNA was stained with DAPI (blue). Scale bars indicate 10 μm. (C) Representative immunoblots treated as in B. Cells were lysed and immunoblotted with anti-GFP (Aurora-C), anti-Aurora-B, anti-INCENP, anti-Survivin, and anti-β-actin antibodies. The experiments were repeated three times with three biological replicates and the figures show representative images.

To characterize the structural interactions of Aurora-C and -B with INCENP and Survivin in the CPC, and investigate if active forms of both Aurora-C and -B along with INCENP and Survivin are necessary for proper localization of the CPC in the cell, we performed co-immunoprecipitations and subcellular localization studies following RNA interference-mediated depletion of individual proteins. Immunoprecipitation from PC-3 cells, which express relatively higher levels of endogenous Aurora-C revealed the presence of INCENP and Survivin in each of the two kinase-complexes but not coexistence of the two kinases in the same complex despite markedly lower relative amount of Aurora-C complex with Survivin and INCENP compared with the Aurora-B complex (Fig 2A). Similar results were also observed with SK-OV-3 and MCF-10A cells (data not shown). Absence of either Aurora-C or -B did not interfere with localization or stability of the other kinase and their respective complex with INCENP and Survivin, but knocking down INCENP or Survivin resulted in reduction of all the remaining proteins in the complexes (Figs 2A–2C). Interestingly, siRNA silencing of CPC component in PC-3 cells showed that depletion of one of the CPC components caused reduction of protein stability of all others (S2 Fig).

**Fig 3:**
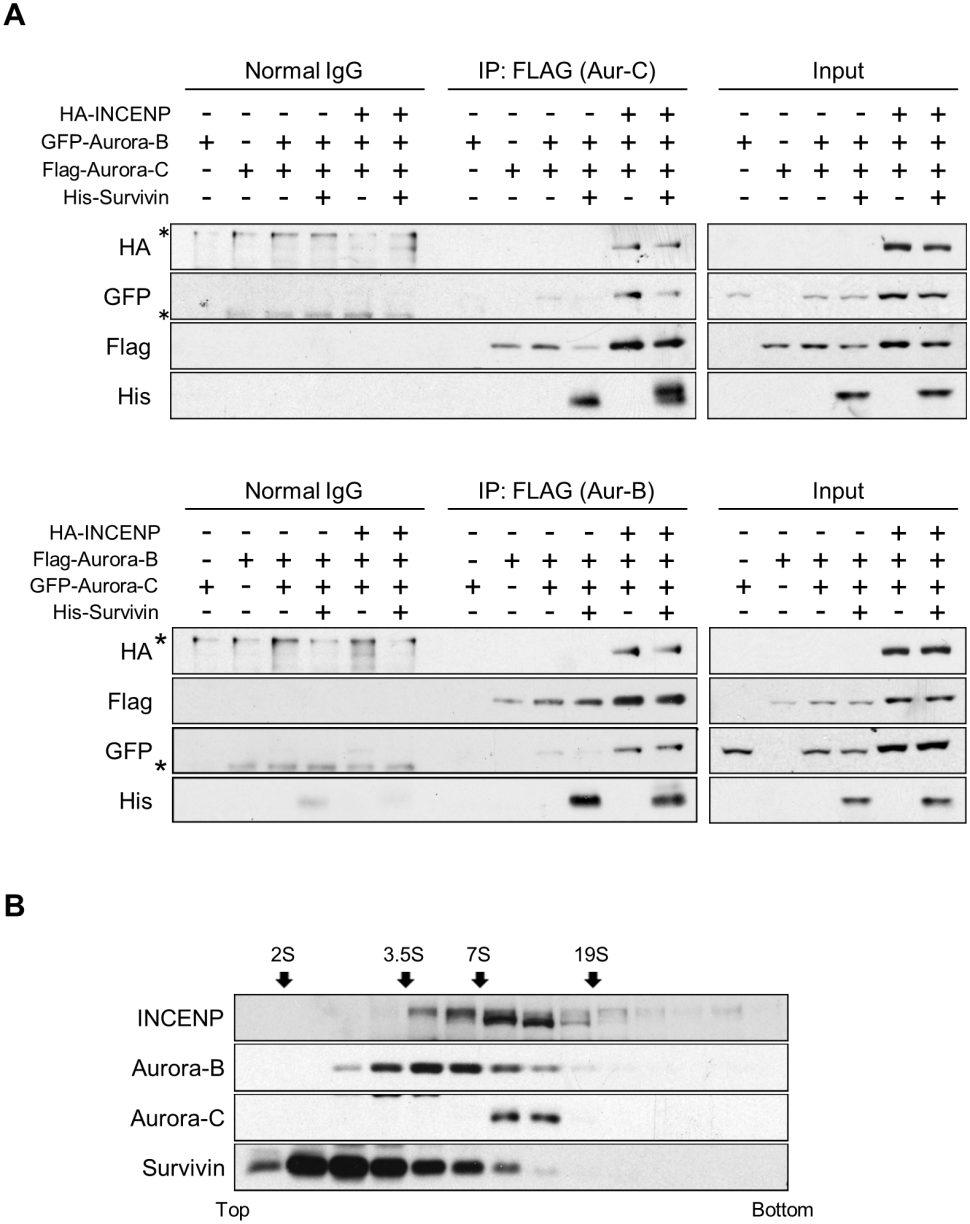
Aurora-C and Aurora-B Complexes in immune precipitates and in Sucrose Density Gradients. (A) 293T cells were transfected with HA-INCENP, Flag-Aurora-C, GFP-Aurora-B, and His-Survivin (top panel). Flag-Aurora-B and GFP-Aurora-C were co-transfected with HA-INCENP and His-Survivin in 293T cells (bottom panel). 24 h after transfection, cells were lysed and immunoprecipitated by anti-Flag antibody or normal mouse IgG. Immunoprecipitates were immunoblotted with indicated antibodies. Absence or presence of each protein are indicated with (-) and (+) signs, respectively. Asterisks indicate non-specific bands. (B) Nocodazole-treated SK-OV-3 cell lysate was sedimented on 5–30% sucrose density gradients. Equal volumes of each fraction were run on SDS-PAGE gels and immunoblotted with anti-INCENP, anti-Aurora-B, anti-Aurora-C, and anti-Survivin antibodies. Thyroglobulin (19S), γ-globulin (7S), ovalbumin (3.5S) and myoglobin (2S) were sedimented in a parallel gradient as markers. Each experiment was repeated twice with three biological replicates and the figures show representative images.

Since results from RNA interference and immunoprecipitation experiments suggested the existence of separate Aurora-C and -B complexes, we investigated if such distinct complexes can be detected following co-expression of transfected Aurora-C and -B in the presence or absence of INCENP and Survivin proteins. Immunoprecipitation experiments of cells ectopically co-expressing Aurora-C and -B and also Survivin detected weak association of the two kinases in the same immune complex. However, when co-expressed with INCENP, Aurora-C, and -B were readily detectable in the same immune complex with increased amount of both Aurora-C and -B in the presence of INCENP (Fig 3A). In order to resolve structural integrity of the Aurora-C and -B complexes *in vivo*, we performed sucrose gradient sedimentation of the cell extracts. The results revealed predominant and exclusive presence of Aurora-B complex in lower density gradients compared with the Aurora-C complex. The two complexes also showed co-sedimentation in gradients of higher density associated with relatively more abundant INCENP (Fig 3B). These findings indicate that although Aurora-B exists in a complex exclusive of Aurora-C, the two kinases may become part of a larger complex due to oligomerization mediated by INCENP.

### Regulation of Aurora-C Interaction with INCENP and Activation

**Fig 4:**
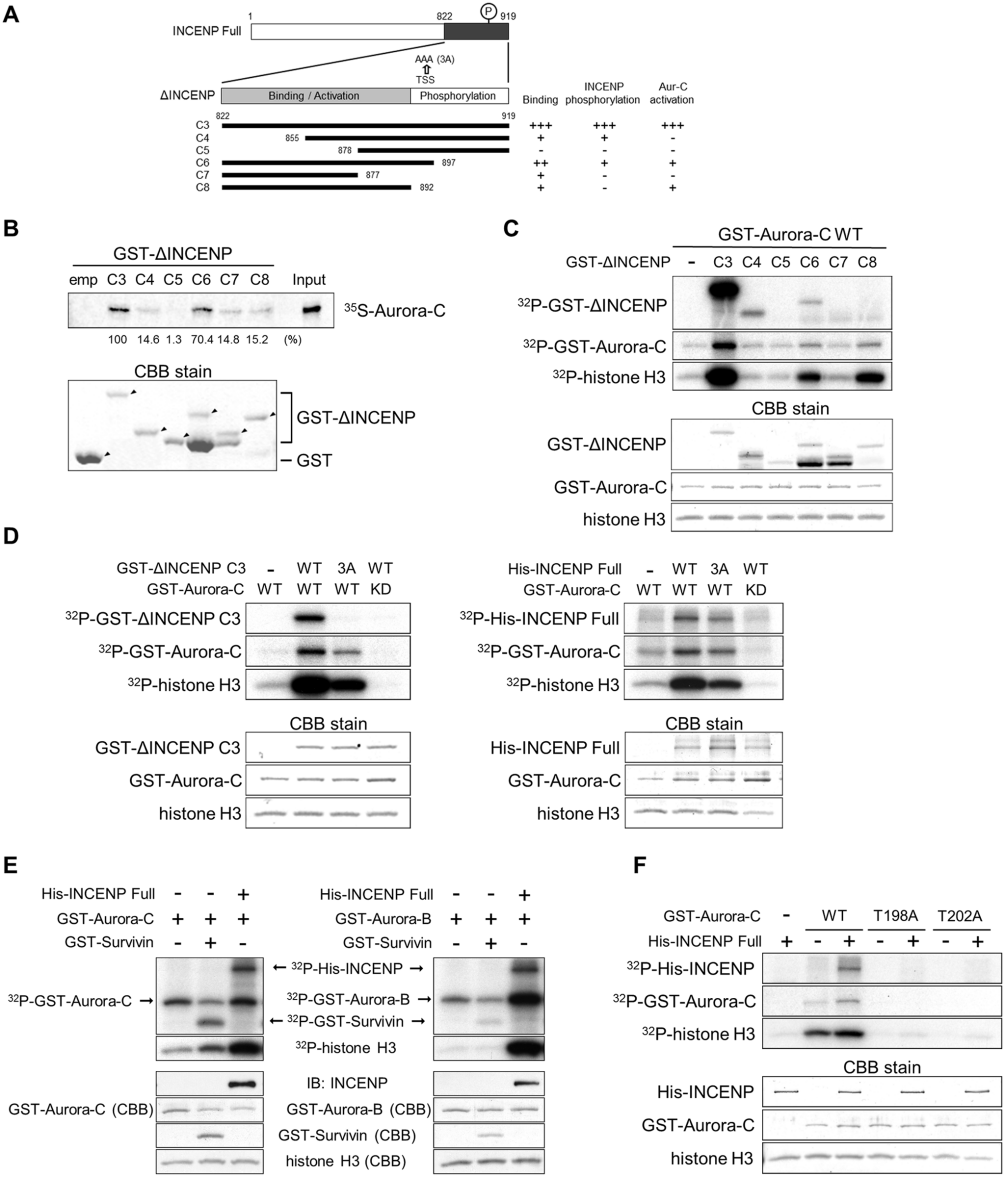
Direct binding and phosphorylation dependent Aurora-C activation through conserved IN box domain of INCENP. (A) Schematic represents INCENP full-length and carboxyl terminal deletion mutants (C3-C8) [5]. The deduced regions of INCENP involved in Aurora-B and Aurora-C binding (aa 822–897) and activation (aa 822–892), as well as the domain serving as a substrate for phosphorylation (aa 893–919) are indicated. 3A indicates three alanine mutants of TSS motif (T893, S894, S895) for Aurora-B and Aurora-C phosphorylation sites. (B) ^35^S-labeled *in vitro* translated Aurora-C was incubated with beads bound to either GST or GST-ΔINCENP deletions. Bound Aurora-C and GST-ΔINCENP deletions were visualized by autoradiography and CBB staining, respectively. Relative Aurora-C binding to GST-INCENP deletions was measured and quantified by ImageJ software. Arrowheads show GST fusion proteins. (C) GST-Aurora-C was incubated with GST-ΔINCENP deletions and histone H3 *in vitro*. Protein amount was determined by CBB stain and kinase activity was measured using histone H3 as substrate. [γ^32^P]-ATP incorporation in proteins was visualized by autoradiography. (D) GST-Aurora-C WT or KD mutants were incubated with histone H3, either WT or 3A mutant of GST-ΔINCENP C3 (left panel) or full length His-INCENP (right panel) *in vitro*, and analyzed as in C. (E) GST-Aurora-C or GST-Aurora-B was co-infected with His-INCENP and GST-Survivin in Sf-9 cells and proteins were isolated on glutathione sepharose. Proteins were detected by immunoblot with anti-INCENP antibody or CBB stain, and kinase activity was measured using histone H3 as substrate as in C. Absence or presence of each protein are indicated with (-) and (+) signs, respectively. (F) GST-Aurora-C WT, T198A or T202A mutants were incubated with full-length His-INCENP and histone H3 *in vitro*, and analyzed as in C. Experiments were repeated twice and representative images are shown in each figure.

In view of the above observations and prior findings on the roles of INCENP and Survivin in Aurora-B activation [5, 26, 27], we next examined if INCENP and Survivin binding and phosphorylation influence activation of Aurora-C and the stability of the proteins in the complex. We and others have earlier reported that the C-terminal end of INCENP spanning the IN box interacts with Aurora-C and is indispensable and sufficient for Aurora-C activation [11, 12]. To fine map the domains within the C-terminal end of INCENP involved in binding and possibly activation of Aurora-C, a series of INCENP deletion mutants from the C-terminal end, characterized in detail for their binding and activation of Aurora-B [5] were assayed for their binding with and phosphorylation as well as activation of Aurora-C (Fig 4A). *In vitro* binding experiments revealed that the C3 fragment (822–919), lacking the coiled coil region but containing the IN box interacted with and activated Aurora-C while also being phosphorylated by the kinase. The fragments C4 (855–919) and C5 (878–919) induced almost no or minimal activation while displaying very weak binding to Aurora-C with C4 being moderately phosphorylated. Among the remaining three fragments, C6 (822–897) showed much stronger interaction than C7 (822–877) and C8 (822–892), while C6 and C8 activated Aurora-C, albeit less than C3 (Fig 4B and 4C). These findings were similar to those reported earlier for Aurora-B activation by INCENP [5]. We next evaluated whether or not the TSS motif (T893, S894, S895) within the IN box domain of INCENP is involved in Aurora-C activation. *In vitro* kinase assays of the C3 fragment and the full length INCENP with either the WT or AAA mutant (3A) revealed that the TSS motif is the target phosphorylation site of Aurora-C, which when phosphorylated stimulates kinase activity of Aurora-C (Fig 4D). Absence of INCENP phosphorylation and lack of any kinase activity in presence of the catalytically inactive mutant of Aurora-C in the control reactions ruled out involvement of any contaminant insect kinase and confirmed the validity of the results. Similar phosphorylation of INCENP and activation by both Aurora-B and -C kinases separately co-expressed with INCENP in human 293T cells and assayed with precipitated immune complexes *in vitro* further documented that INCENP phosphorylation activates both kinases in a similar manner (S3A Fig). *In vitro* kinase assays on pulled down protein complexes from Sf-9 cells co-infected with WT or 3A mutant of INCENP together with the Aurora-B and -C kinases also confirmed the role of the TSS motif phosphorylation of INCENP in the activation of the two kinases (S3B Fig).

In view of the fact that endogenous Survivin was found to be in the Aurora-C complex, we investigated the relative involvement, if any, of Survivin as a substrate and/or activator of Aurora-C. Phosphorylation of Survivin with simultaneous activation of Aurora-C was observed in the GST tagged Aurora-C complex pulled down from Sf-9 cell lysate co-infected with Aurora-C and Survivin constructs. The levels of Survivin phosphorylation and Aurora-C activation were, however, lower compared with those detected in case of INCENP mediated activation of Aurora-C. Interestingly, Aurora-B revealed minimal phosphorylation of Survivin and no activation of its kinase activity in identical assays performed with recombinant proteins expressed in Sf-9 cells (Fig 4E). Similarly, i*n vitro* reconstituted kinase assays with recombinant INCENP, Aurora-C, and Survivin proteins expressed in Sf-9 cells in the presence of histone H3 revealed that Survivin is phosphorylated by Aurora-C with simultaneous up-regulation of Aurora-C kinase activity (S3C Fig) albeit at a lower level than what is observed for INCENP in similar pull down experiments (compare S3C Fig and Fig 4E). Since two Threonine residues, T171 within a putative PKA phosphorylation site in the activation loop and an adjacent T175 of mouse Aie1/Aurora-C, have earlier been reported to be critical for enzyme activity [28], we investigated if the corresponding T198 and T202 of the human Aurora-C are also essential for activation of the human enzyme. *In vitro* kinase assays of T198A and T202A in the presence of INCENP revealed involvement of both residues in activation of the human Aurora-C kinase (Fig 4F). These findings were further corroborated by immune complex kinase assay with 293T cells expressing transfected Flag tagged WT, T198A, or T202A Aurora-C mutants together with either WT or INCENP 3A mutant proteins. The 3A mutant of INCENP caused distinctly lowered Aurora-C phosphorylation and activation compared to WT. As expected, the T198A and T202A Aurora-C mutants revealed complete loss of phosphorylation and lack of activation in the presence of either WT or the 3A mutant of INCENP (S3D Fig).

### Aurora-C Phosphorylation Dependent Protein Stabilization of INCENP

**Fig 5:**
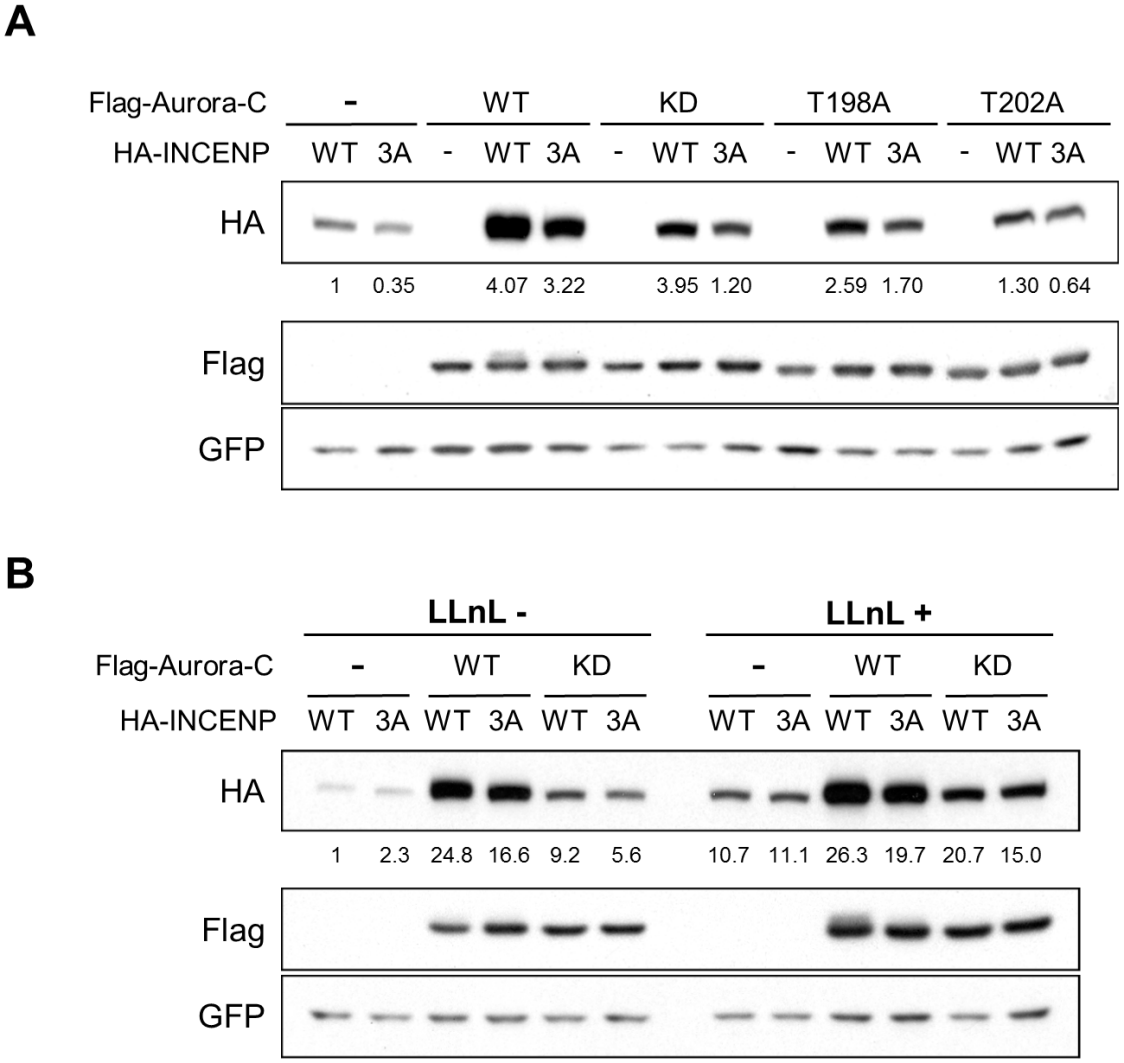
Stabilization of INCENP by Aurora-C phosphorylation. (A) Flag-Aurora-C WT, KD, T198A, or T202A mutants were co-transfected with HA-INCENP WT or 3A mutant and GFP empty vector in 293T cells. After 24 h transfection, cells were analyzed. GFP was used as internal control for transfection. Quantification of HA-INCENP protein levels were determined by using the ImageJ software. Relative fold change with normalization by GFP expression is shown. (B) 24 h after transfection, 293T cells were split and grown in culture for additional 24 h. Then cells were treated with 50 μM LLnL for 6 h and analyzed as in A. Experiments (A) and (B) were repeated three times with two biological replicates and figures show the representative results.

Since depletion of Aurora-C and -B by RNA interference revealed reduced localization at inner centromere and also lowered INCENP levels in the western blots (Fig 2B and S2 Fig), we investigated if Aurora-C binding and/or phosphorylation influence stability of INCENP. To address the issue, steady state levels of INCENP were assayed in 293T cells transiently transfected with Flag tagged Aurora-C and HA tagged INCENP constructs along with GFP as an internal control. WT or 3A mutants of INCENP were expressed either in the absence or in the presence of WT and kinase inactive K72R, T198A, and T202A mutants of Aurora-C. In the presence of Aurora-C, steady state level of INCENP was higher, though the intracellular concentration also appeared to be a function of its phosphorylated state. Thus, INCENP amount was most elevated when the WT protein was expressed in the presence of WT Aurora-C, but appeared lower for the unphosphorylatable 3A mutant (Fig 5A). INCENP amount of both WT and 3A mutant were further reduced when co-expressed with the kinase inactive Aurora-C mutants (Fig 5A). The results suggested that Aurora-C phosphorylation influences the intracellular stability of INCENP. Aurora-B also had similar effects on INCENP stability (data not shown). To investigate if Aurora-C-dependent stability of INCENP is influenced by proteasomal degradation, we compared the INCENP steady state levels in 293T cells co-expressing the WT or mutant Aurora-C in the absence or presence of proteasomal inhibitor N-acetyl-L-leucyl-L-leucyl-L-norleucinal (LLnL). The data revealed that reduced stability of un-phosphorylated INCENP is partly dependent on increased susceptibility to proteasomal degradation since in the presence of LLnL the steady state levels of the protein appeared higher than in its absence in parallel experiments (Fig 5B).

### Survivin Phosphorylation by Aurora-C and -B in Chromosome Alignment, Segregation and Cytokinesis

**Fig 6:**
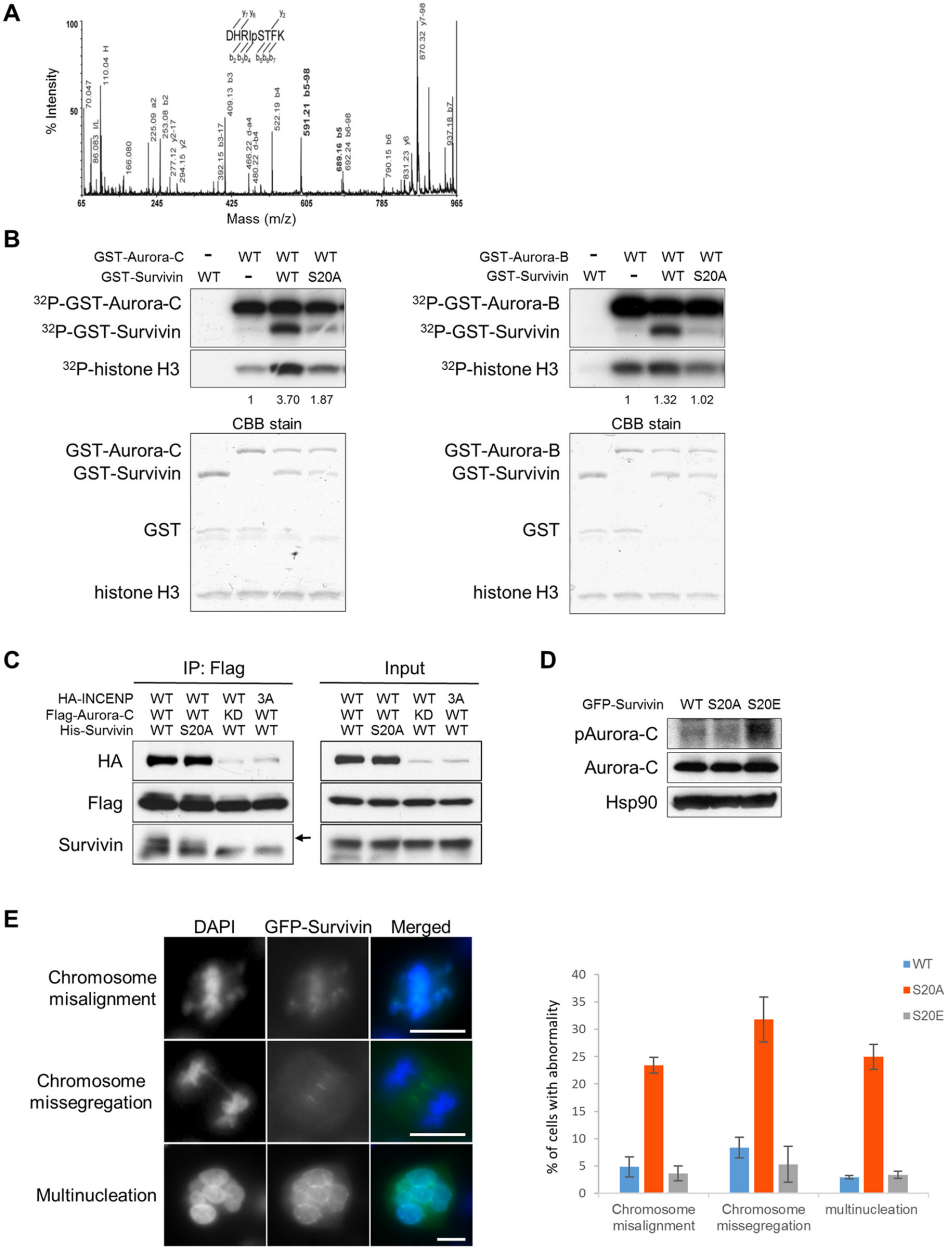
Aurora-C phosphorylates Survivin on Serine 20. (A) Fragmentation of DHRIpSTFK on the TofTof. Inset summarizes ions found. A key ion is the b5, mainly observed as the -98 neutral-loss ion (loss of phosphoric acid), indicating it as the primary site of phosphorylation. Essentially complete sequence ion coverage was observed considering both “b” and “y” type ions. Detection of the Ile-specific d4 fragment ions provides confirmation that the overall sequence assignment is correct. (B) Sf-9 cells were co-infected with GST-Survivin WT or S20A mutant and GST-Aurora-C WT or KD (left panel) or GST-Aurora-B WT or KD (right panel). 48 h after infection, proteins were isolated on glutathione sepharose, co-precipitated GST-Aurora-C, GST-Aurora-B, and GST-Survivin were incubated with [γ^32^P]-ATP and histone H3 as substrate. The reactions were resolved by SDS-PAGE and visualized by autoradiography (top) or CBB stained (bottom). Relative [γ^32^P]-ATP incorporation to histone H3 shown was normalized by total histone H3 and quantified by ImageJ software. Experiments were repeated three times and showed reproducible results. (C) 293T cells were co-transfected with HA-INCENP, Flag-Aurora-C, and His-Survivin plasmids respectively. 24 h after transfection, cells were lysed and immunoprecipitated by anti-Flag antibody. Immunoprecipitates were immunoblotted with indicated antibodies. Arrow indicates slower mobility sift band of Survivin. (D) HeLa cells stably expressing GFP-Survivin WT, S20A, or S20E mutant were treated with 40 ng/ml nocodazole for 16 h. Cells were lysed and immunoblotted with indicated antibodies. (E) Analysis of cellular and mitotic defects in HeLa cell clones stably expressing GFP-Survivin WT, S20A, or S20E mutant. Representative images of GFP-Survivin S20A expressing cells with abnormal chromosome alignment, abnormal chromosome separation, and multinucleation are shown on the left. Green fluorescence shows distribution of Survivin and DNA is stained with DAPI (blue). The percentages of chromosome misalignment, chromosome missegregation, and multinucleated giant abnormal cells in the indicated transfected clones are shown in the bar graph on the right. At least 200 cells in each of the clones were analyzed for this assay. Data represent mean values ± SD from two clones (n = 2). Scale bar indicates 10 μm. Experiments were repeated twice with two biological replicates in each experiment. Figures show the representative images.

In view of the finding that Aurora-C phosphorylation of Survivin positively regulates Aurora-C kinase activity, we mapped the phosphorylation site by mass spectrometry of *in vitro* phosphorylated Survivin peptides and also by assaying for Aurora-C phosphorylation of Survivin site directed mutants *in vitro* and *in vivo*. The MALDI spectrum of the phosphorylated Survivin digest exhibited MH+ signals corresponding to both the phosphorylated and the unmodified peptide (data not shown). The observed mass difference, 79.953, was in good agreement with the expected mass shift for phosphorylation of 79.966. The spectrum of phosphorylated peptide revealed complete sequence coverage including the neutral loss ions (-98) indicating elimination of H_3_PO_4_ from some of the b-ions. The presence of both the intact b5 (at 689.3 m/z) and its neutral-loss fragment (b5-98, at 591.3 m/z) pointed to the primary site of phosphorylation as Serine 20 of Survivin, which falls within one of the consensus Aurora kinases phosphorylation motifs RXS/T or RXXS/T (Fig 6A). To validate the finding, Serine 20 was mutated to Alanine and utilized along with Aurora-C WT proteins expressed in Sf-9 cells for in vitro kinase assays. Histone H3 was included in the reactions as a substrate of Aurora-C to detect the effects of Survivin phosphorylation on Aurora-C kinase activity. Wild type Survivin revealed clear phosphorylation in presence of Aurora-C that was markedly reduced in the S20A mutant. The level of Survivin phosphorylation correlated with Aurora-C kinase activity revealed by histone H3 phosphorylation (Fig 6B). Interestingly, Aurora-B also phosphorylates Serine 20, but this phosphorylation, unlike in case of Aurora-C, did not appear to activate Aurora-B kinase activity (Fig 6B). Aurora-C phosphorylation of Survivin was also indicated by the presence of slower mobility Survivin bands in the Aurora-C immune complexes isolated form 293T cells co-expressing Aurora-C, INCENP, and Survivin. The slower mobility Survivin band was markedly reduced following treatment with λ Protein Phosphatase (S4 Fig) and in the immune complex containing the S20A mutant, suggesting that Serine 20 phosphorylation is responsible for the slower mobility band detected in the Aurora-C immune complex. This finding was additionally confirmed by the absence of slower mobility Survivin bands in the immune complexes containing either an Aurora-C KD mutant or those with a minimally activated Aurora-C kinase in presence of the IN box 3A mutant of INCENP (Fig 6C). To investigate the functional significance of Survivin Serine 20 phosphorylation, we generated stable clones of HeLa cells expressing GFP tagged wild type Survivin as well as non-phosphorylatable S20A and phosphor-mimic S20E Survivin mutants. A total of about forty clones of each were isolated and verified for the expression of the transgene by western blot analysis with the anti-GFP antibody and by RT-PCR analysis for the transgene specific transcript. Fifteen to twenty percent of the selected clones were found to express the transgenes. Consistent with the result of *in vitro* kinase assay, Aurora-C kinase activity was lower in the S20A expressing clones compared to WT expressing clones and was significantly enhanced in the S20E expressing clones (Fig 6D). Interestingly, the S20A expressing clones frequently revealed marked changes in cell morphology with progressively increasing number of cells becoming rounded and floating off in the culture medium. The S20A expressing clones could be maintained only for about five passages on average, while the WT and S20E expressing clones continued to grow, as expected. Two clones of each transgene, GFP-WT, GFP-S20A, and GFP-S20E, expressing cells were analyzed in greater detail for cellular, nuclear and mitotic anomalies. A significantly high number of S20A expressing cells were found to be of giant morphology with multiple nuclei and mitotic abnormalities reflected in the form of misaligned and missegregating chromosomes. Quantitative analyses revealed that about twenty five percent of the S20A expressing cells had chromosome misalignment, chromosome missegregation, or multinucleation compared with only about two to four percent of the WT and the S20E expressing cells showing such abnormalities (Fig 6E). The results thus clearly demonstrated that Survivin phosphorylation on Serine 20 plays an important role in the regulation of proper chromosome alignment, segregation, and possibly cytokinesis.

## Discussion

Aurora-C kinase is a closely related paralogue of Aurora-B kinase [29, 30]. Like Aurora-B, Aurora-C has been implicated in the CPC functions in somatic cells based on studies with ectopically expressed transgene in cell lines grown *in vitro* [11, 12]. Nevertheless, these observations have remained somewhat inconclusive in view of the very low or undetectable levels of endogenous Aurora-C detected in most somatic tissues. However, recent findings on functional involvement of Aurora-C in the CPC during human pre-implantation embryo development [14] and early embryonic development of mouse [15] have helped resolve the issue to some extent. Additionally, the observation that Aurora-C can sustain CPC function during early development in *AURKB* null mouse embryos till implantation stage [31] reinforces the fact that Aurora-C is the major CPC kinase during early embryonic cell divisions *in vivo*. The findings that Aurora-C expression is elevated in multiple human cancers indicates a functional role for the kinase in adult somatic tissues and makes it imperative that the molecular details of Aurora-C interactions with the CPC components and the functional consequences of Aurora-C over expression in cancer cells be well elucidated. It is relevant in this context that we have recently reported transcriptional down-regulation of *AURKC* locus due to hyper-methylation of CpG islands in the promoter to be primarily responsible for low expression of Aurora-C in adult somatic tissues and cell lines *in vitro* [32]. Our study further revealed that CpG hypo-methylation besides down-regulation of the previously published transcriptional repressor PLZF/ZBTB16 [33] is associated with elevated expression of Aurora-C in human cancer cells, also validated in The Cancer Genome Atlas (TCGA) data set across different human cancers. These findings imply that functional interactions of Aurora-C with CPC proteins may be critically influencing the phenotype of Aurora-C overexpressing human cancers due to possible effects on chromosome segregation and consequential induction of genomic instability.

The findings of this investigation reveal that Aurora-C-Survivin-INCENP passenger protein complex in addition to sharing some of the evolutionarily conserved structural and functional interaction characteristics with Aurora-B complex also displays its own distinct features. The identification of Aurora-C and -B in the same complex was reported in earlier studies utilizing ectopically expressed transfected Aurora-C [12, 20, 34]. However, our finding that RNA interference mediated silencing of either Aurora-C or -B does not affect the localization of the other kinase despite influencing protein stability of both proteins argues against the two active kinases being necessary for proper targeting of the individual complexes. Sucrose gradient sedimentation results also suggest existence of two independent Aurora-C and -B CPC. Reciprocal co-immunoprecipitation of endogenous Aurora-C and -B with respective antibodies further showed that these two members of the Aurora kinase family exist in two separate complexes. Co-sedimentation of the two kinase complexes in higher sucrose gradients, however, indicates that Aurora-C and -B may become part of a larger ternary complex involving INCENP, as described for ternary sub-complex containing Survivin, INCENP, and Borealin [35]. This scenario may partly explain why loss of any CPC component might influence protein stability of both CPC complexes and ultimately give rise to similar phenotypic abnormalities during mitotic progression.

Activation of Aurora-C involves the conserved Threonine 198 and Threonine 202 residues in the activation loop of the catalytic domains. While the role of Threonine 198 in activation of Aurora-C [12] is known, the role of the conserved Threonine 202 in activation remains to be determined. It is plausible that this residue plays a critical role in the development of proper structural conformation required for Threonine 198 phosphorylation mediated activation of the enzyme.

Finally, it is significant that despite sharing multiple functional interaction characteristics with Aurora-B in mitotic cells, phosphorylation of Survivin on Serine 20 appears to be involved in the activation of Aurora-C not evident in the case of Aurora-B kinase. Though it does not appear to be involved in subcellular targeting of the protein, as documented for Aurora-B mediated Threonine 117 phosphorylation on Survivin [36], results of this study provide evidence in favor of Serine 20 phosphorylation playing an important role in regulating chromosome segregation and cytokinesis. Based on our finding of increased incidence of aberrant chromosome alignment, segregation and multi-nucleation in the S20A expressing cells, it seems possible that this phosphorylation may be playing a role in spindle checkpoint activation and/or cytokinesis by regulating recruitment of additional proteins. Since Serine 20 of Survivin is within the BIR domain that is essential for spindle checkpoint function [37], such possibility is worthy of investigation. Additionally, it may also be interesting to investigate if Serine 20 phosphorylation of Survivin influences K63 linked ubiquitination of the protein, which was reported to be involved in regulating chromosome segregation [38].

**Fig 7:**
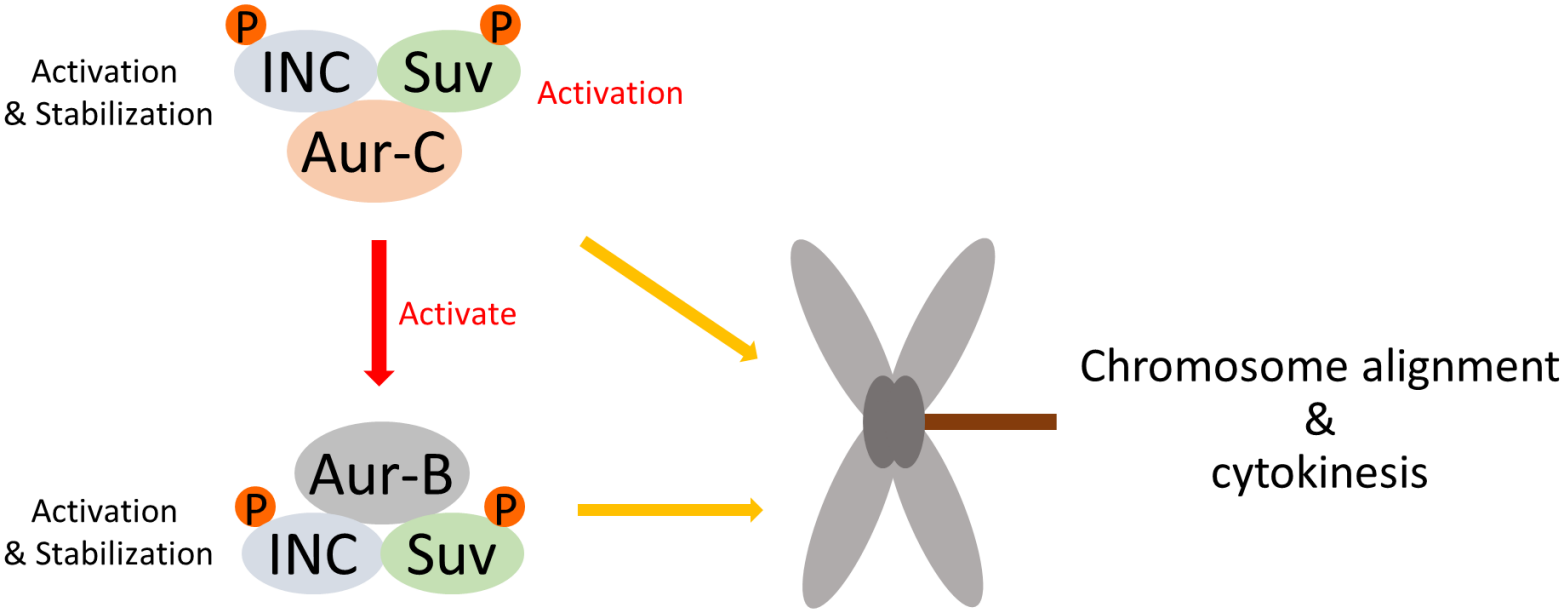
Hypothetical scheme of functional roles for Aurora-C and Aurora-B CPC.

In summary, this study demonstrates that endogenous Aurora-C in human cancer cells besides existing in independent passenger protein complexes distinct from Aurora-B may also form larger ternary complex with Aurora-B. Aurora-C and -B kinase interactions with INCENP facilitate stabilization of INCENP and activation of their kinase activities. Phosphorylation of Survivin on Serine 20 by Aurora-C and -B kinases appears to be involved in the activation of Aurora-C and play an important role in regulating mitotic chromosome alignment, segregation and possibly cytokinesis (Fig 7).

## Supporting Information

S1 Fig Aurora-C KD mutant cause mis-localization of INCENP, Survivin and Aurora-B. Tet-on HeLa GFP-Aurora-C WT and KD cells treated with Dox for 48 h were immunostained with (A) anti-α/β-tubulin (red). DNA was stained with DAPI (blue). (B; top) Tet-on HeLa GFP-Aurora-C KD cells in the presence or absence of Dox treatment were immunostained with anti-INCENP, (B; middle) Survivin or (B; bottom) Aurora-B antibodies (red). Scale bar indicates 10 μm.
(TIF)

S2 Fig Aurora-C and Aurora-B mutually control their protein stability. PC3 cells transfected with control siRNA (GL2) or with siRNA against Aurora-C, Aurora-B, INCENP, or Survivin for 30 h were treated with nocodazole for 16 h. Cells were lysed and immunoblotted with anti-Aurora-C, anti-Aurora-B, anti-INCENP, anti-Survivin, and anti-β-actin antibodies.
(TIF)

S3 Fig Phosphorylation of IN box in INCENP is essential for Aurora-C activation. (A) Flag-Aurora-B or Flag-Aurora-C was co-transfected with either empty vector or full-length HA-INCENP WT into 293T cells and immunoprecipitated with anti-Flag antibody. Kinase activity was measured using histone H3 as substrate. [γ^32^P]-ATP-marked proteins were visualized by autoradiography (upper panel). Immunoprecipitates were immunoblotted with anti-Flag or anti-HA antibodies. Histone H3 was visualized by ponceu staining (bottom panel). (B) GST-Aurora-C or GST-Aurora-B was co-expressed with full-length His-INCENP WT or 3A mutant in Sf9 cells and proteins were isolated on glutathione sepharose. Proteins were detected by immunoblot with anti-INCENP antibody or CBB stain, and kinase activity was measured using histone H3 as substrate as in A. (C) GST-Aurora-C WT was incubated with either full-length His-INCENP or GST-Survivin and histone H3 *in vitro*, and analyzed as in A. (D) Flag-Aurora-C WT, T198A, or T202A mutants were co-transfected with either empty vector or full-length HA-INCENP WT or 3A mutant into 293T cells and immunoprecipitated with anti-Flag antibody. Kinase activity was measured using histone H3 as substrate, and analyzed as in A.
(TIF)

S4 Fig The phosphorylation of Survivin Ser20 by Aurora-C. 293T cells were co-transfected with HA-INCENP, Flag-Aurora-C, and His-Survivin mutants. 24 h after transfection, cells were lysed and immunoprecipitated by using anti-Flag antibody. The immune complexes were incubated with 20 units of λPPase (New England Biolabs) or glycerol in the λPPase buffer for 30 min at 30°C. The reaction was terminated by adding SDS sample buffer and resolved by SDS-PAGE.
(TIF)
