# Supplementary figures and images for: Aurora-C Interactions with Survivin and INCENP Reveal Shared and Distinct Features Compared with Aurora-B Chromosome Passenger Protein Complex
Source: PLoS One. 2016 Jun 22;11(6):e0157305. doi: 10.1371/journal.pone.0157305 (PMC4917241; doi:10.1371/journal.pone.0157305)

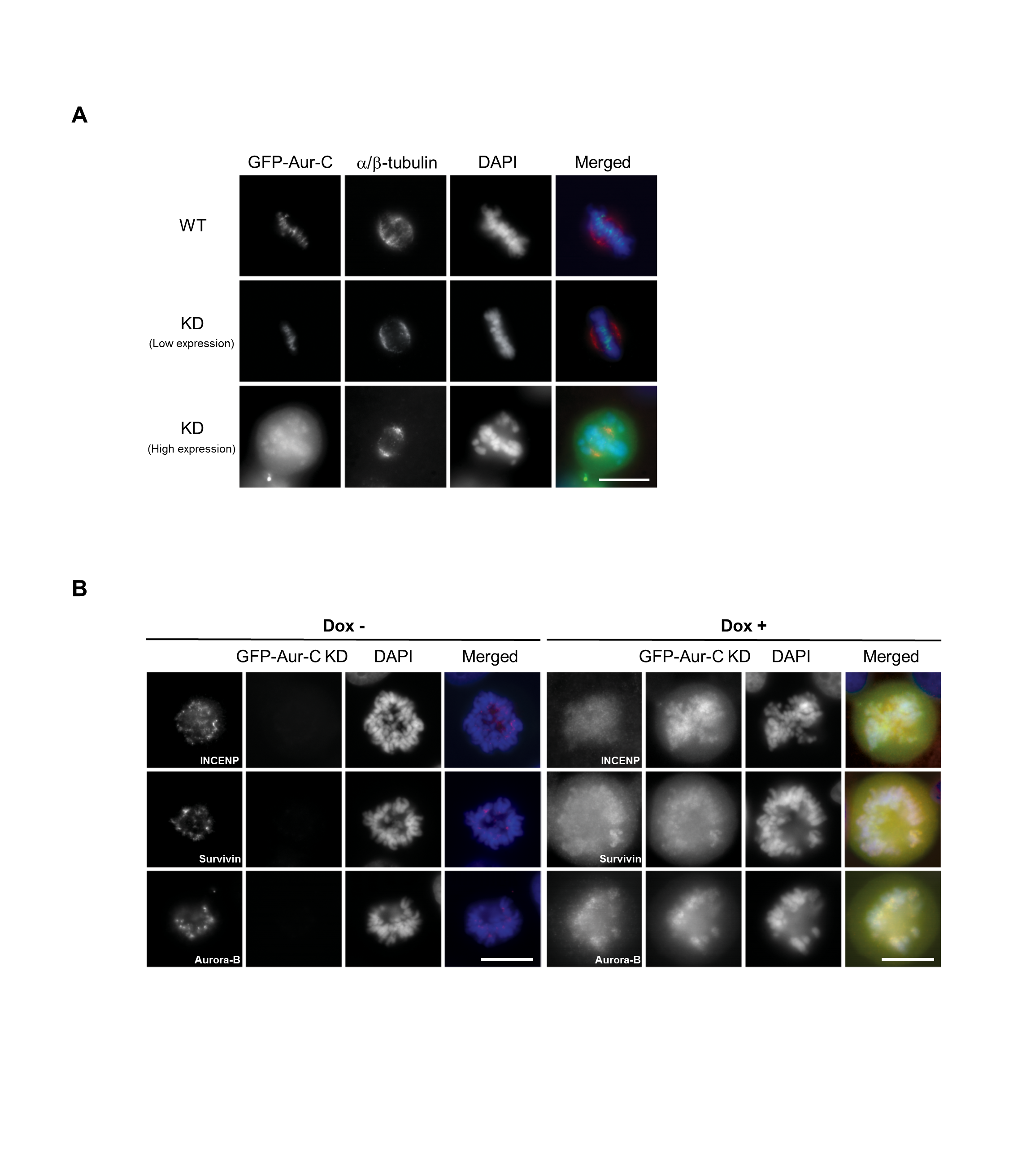

Supplement: S1 Fig — Tet-on HeLa GFP-Aurora-C WT and KD cells treated with Dox for 48 h were immunostained with (A) anti-α/β-tubulin (red). DNA was stained with DAPI (blue). (B; top) Tet-on HeLa GFP-Aurora-C KD cells in the presence or absence of Dox treatment were immunostained with anti-INCENP, (B; middle) Survivin or (B; bottom) Aurora-B antibodies (red). Scale bar indicates 10 μm. (TIF) [file pone.0157305.s001.tif]

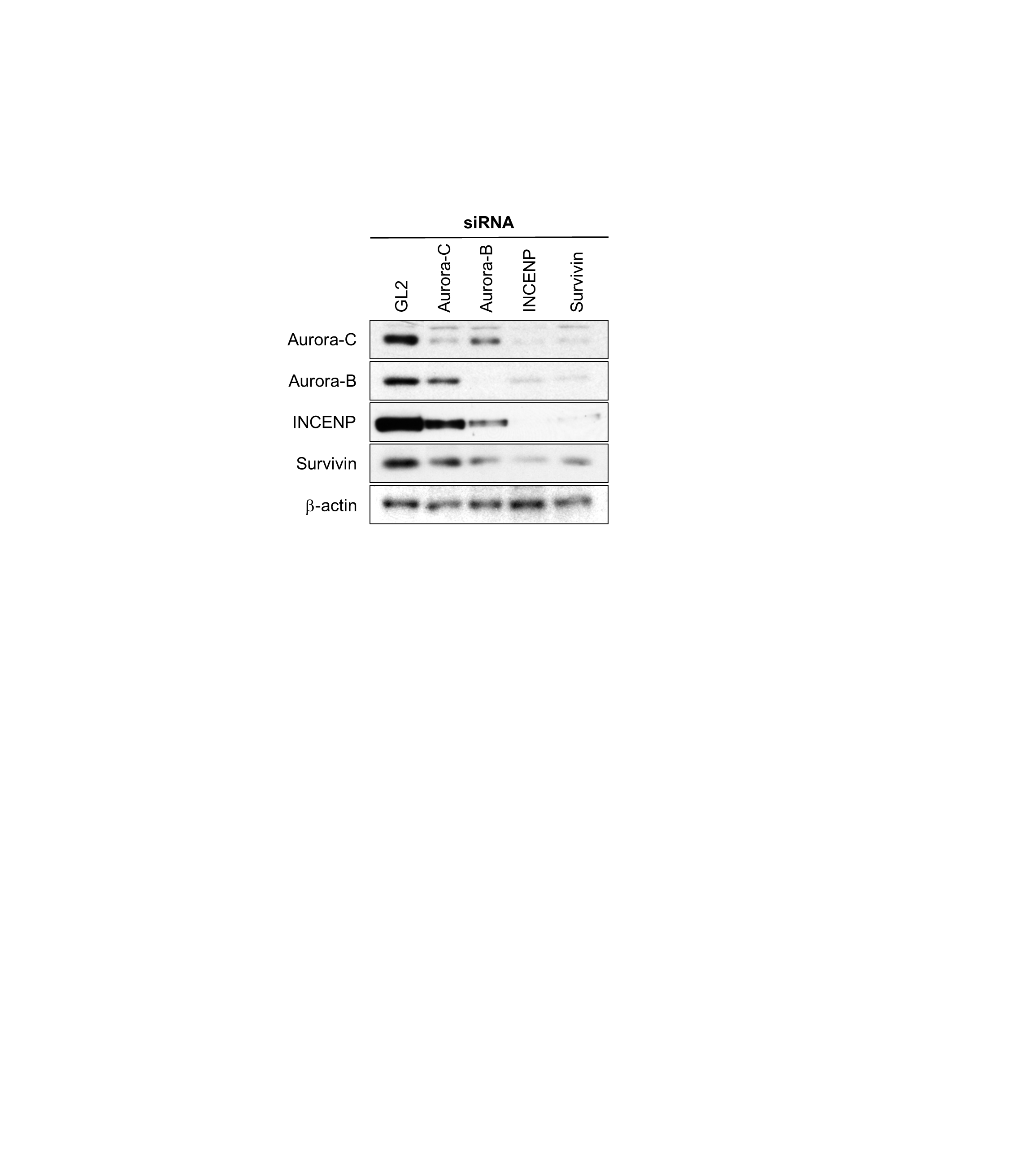

Supplement: S2 Fig — PC3 cells transfected with control siRNA (GL2) or with siRNA against Aurora-C, Aurora-B, INCENP, or Survivin for 30 h were treated with nocodazole for 16 h. Cells were lysed and immunoblotted with anti-Aurora-C, anti-Aurora-B, anti-INCENP, anti-Survivin, and anti-β-actin antibodies. (TIF) [file pone.0157305.s002.tif]

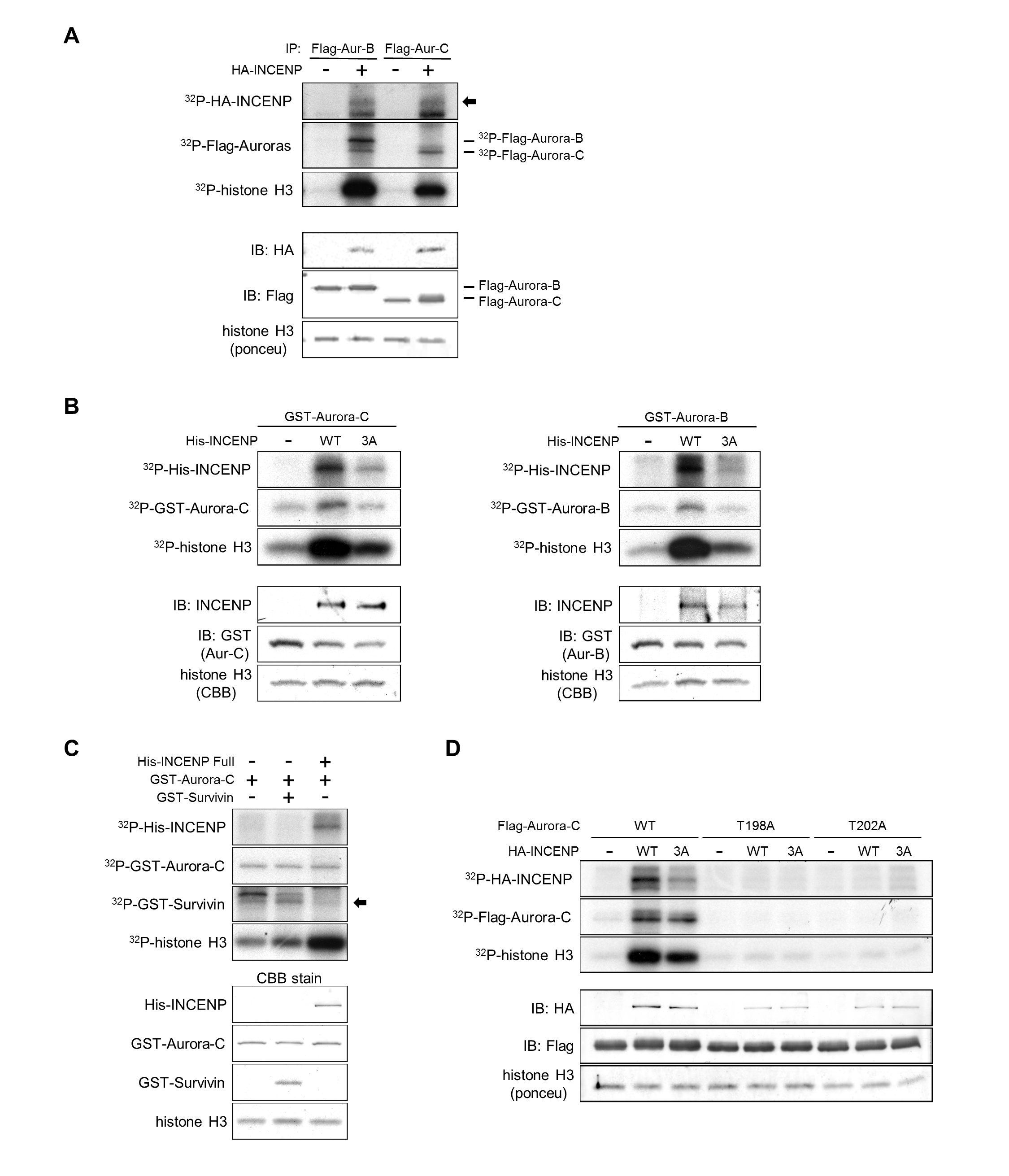

Supplement: S3 Fig — (A) Flag-Aurora-B or Flag-Aurora-C was co-transfected with either empty vector or full-length HA-INCENP WT into 293T cells and immunoprecipitated with anti-Flag antibody. Kinase activity was measured using histone H3 as substrate. [γ32P]-ATP-marked proteins were visualized by autoradiography (upper panel). Immunoprecipitates were immunoblotted with anti-Flag or anti-HA antibodies. Histone H3 was visualized by ponceu staining (bottom panel). (B) GST-Aurora-C or GST-Aurora-B was co-expressed with full-length His-INCENP WT or 3A mutant in Sf9 cells and proteins were isolated on glutathione sepharose. Proteins were detected by immunoblot with anti-INCENP antibody or CBB stain, and kinase activity was measured using histone H3 as substrate as in A. (C) GST-Aurora-C WT was incubated with either full-length His-INCENP or GST-Survivin and histone H3 in vitro, and analyzed as in A. (D) Flag-Aurora-C WT, T198A, or T202A mutants were co-transfected with either empty vector or full-length HA-INCENP WT or 3A mutant into 293T cells and immunoprecipitated with anti-Flag antibody. Kinase activity was measured using histone H3 as substrate, and analyzed as in A. (TIF) [file pone.0157305.s003.tif]

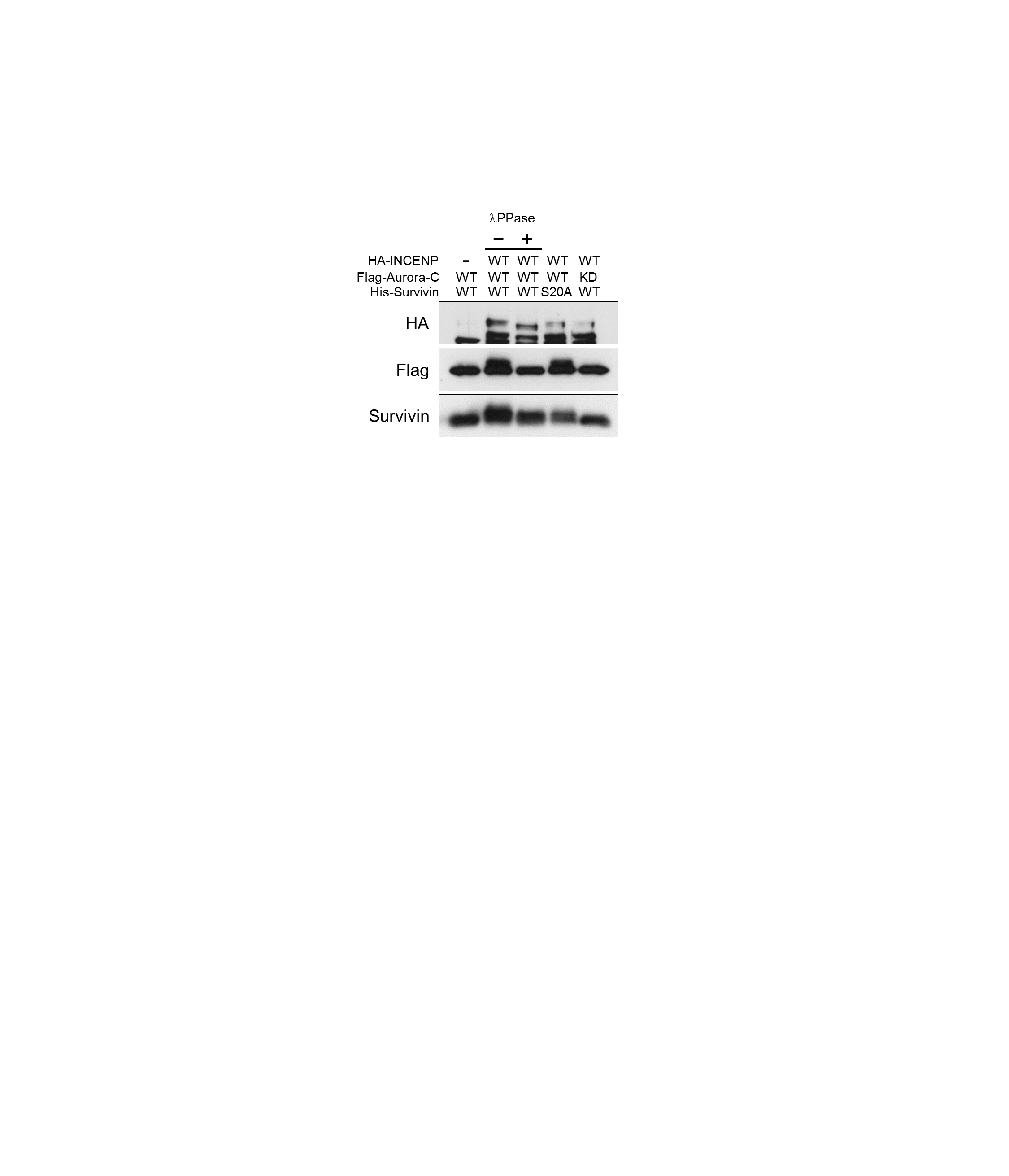

Supplement: S4 Fig — 293T cells were co-transfected with HA-INCENP, Flag-Aurora-C, and His-Survivin mutants. 24 h after transfection, cells were lysed and immunoprecipitated by using anti-Flag antibody. The immune complexes were incubated with 20 units of λPPase (New England Biolabs) or glycerol in the λPPase buffer for 30 min at 30°C. The reaction was terminated by adding SDS sample buffer and resolved by SDS-PAGE. (TIF) [file pone.0157305.s004.tif]
